# Ability to Predict Side-Out Performance by the Setter’s Action Range with First Tempo Availability in Top European Male and Female Teams

**DOI:** 10.3390/ijerph17176326

**Published:** 2020-08-31

**Authors:** Antonio Millán-Sánchez, Manuel J. Parra-Royón, José M. Benítez, Aurelio Ureña Espa

**Affiliations:** 1 Department of Physical Education and Sport, University of Granada, 18011 Granada, Spain; aurena@ugr.es; 2 Department of Computer Science and Artificial Intelligence, DICITS, DaSCI, iMUDS, University of Granada, 18071 Granada, Spain; manuparra@gmail.com (M.J.P.-R.); j.m.benitez@decsai.ugr.es (J.M.B.)

**Keywords:** team sports, quick attack availability, middle blocker, rotation, attack lane, volleyball, performance analysis

## Abstract

The aims of this study were to compare the Setter’s action range with availability of first tempo (SARA) between male and female volleyball; and to determine the relationship between several spatial and offensive variables and their influence in the success of the side-out in male and female volleyball. A total of 1302 side-outs (639 male, 663 female) were registered (2019 European Championship). The ranking, reception efficacy, position and trajectory of the setter between reception and set, first tempo availability, side-out result, rotation, and attack lane were analyzed through Recursive Partitioning for classification, regression and survival tree models and classification and regression trees algorithms. Our results present female teams with more reduced SARAs than male teams, meaning female setters tend to play closer to the net. The correlation between the ranking and the distance from the average position of the setter to the ideal setting zone was not significant. A movement of the setter of 30° or less and more than 1 m in distance might improve the performance of the side-out. Depending on the spatial usage of the setter, some rotations might be more successful than others. When assessing performance, the teams should consider the ability to play quick attacks when their reception is not as precise as they would expect.

## 1. Introduction

The reception efficacy is an accepted and well considered performance indicator in volleyball. Numerous studies have proven its relationship to winning points and matches [1,2]. As a consequence of the sequence of actions in volleyball, the reception efficacy is usually measured through the area in which the setter, responsible for the second contact of the team, performs. Papadimitriou et al. [3] established two setting zones in their study: ideal (zone 2, from the net till 4.5 m) and any other area. Years later, it seemed that a classification in three setting zones was the most frequent: excellent, acceptable, and not acceptable [4,5]. Nevertheless, Ramos et al. [6] classified the setting conditions into three levels, not only based on the setting zone but also on the attack availability: excellent (setter next to the net and all attack options available), reasonable (setter near the net and fewer attack options but still with quick attacks, including the middle blocker), and weak conditions (setter far from the net and only attacks in the wings). More recently, Mercado-Palomino et al. [7] compared the influence in the final ranking of the reception efficacy and the setter’s action range with availability of middle blocker attack (SARA); they found a higher correlation of the latter, although a smaller effect size for the reception efficacy was also presented. Therefore, it seems that throughout the years the way of assessing the ideal setting zone has evolved from an exclusively spatial area to a more complex and dynamic concept.

With a men’s world championship sample, the setter performed from the ideal setting zone 76% of the side-outs [2]. With a similar sample, Mercado-Palomino, Millán-Sánchez, Parra-Royón, Benítez, and Ureña Espa [7] found that the set was carried out in latitude 6 (sixth meter from the left line) with a frequency of 42% and in depth 1 (first meter from the net) with a frequency of 55%. In a comparison between elite (Olympic Games) and national (Portuguese league) female players, elite players displayed more regularity in the setting zone. Furthermore, within the same level of competition, this regularity was higher in the first four classified of the elite level compared to the second four. In the national level, the first four presented higher variability in the space used when setting than the second four [6]. In under-18 Spanish male players, Sánchez et al. [8] studied the relationship between the player that performed the first contact and the setting zone, finding out that the setter performed in the excellent setting zone when the libero hit the first contact and in the acceptable setting zone when it was any other player (*p* < 0.001).

The reception efficacy was found to predict the success in the setting [9,10] and in the consequent attack [1,11], for both male and female teams. Nevertheless, several studies have not been able to prove the relationship between the reception efficacy and the result of the matches in either male or in female players [12,13]. In an overall comparison between men and women, the former presented a higher reception efficacy than the latter, which might be explained by the different height of the net, allowing female players to take more risk in their serves [14].

Concerning the attack zone, several classifications have been made. Some complex divisions consider up to six front row zones and four back row zones [4]. Nevertheless, other authors consider the six official zones established by the Fédération Internationale de Volleyball (FIVB) [6]. Other authors divide the court into attack lanes, regardless of the front or back position of the attacker [15]. In this regard, higher-level teams have shown to be less predictable when it came to choosing the attacker, (i.e., the attack zone was not as dependent on the reception efficacy), but also within the same sample, the best ranked teams presented more variability in the selection of their attack zones than those ranked below [6].

With regard to the position of the players on the court, volleyball is quite a unique sport, given that all six players in the line-up must play in all six zones following the mandatory rotation system, according to which all players have to rotate one zone clockwise whenever they score a point as the receiving team (rule 7.6.2) [16]. Despite high-level players usually specializing in certain zones and actions [17], all of them must comply with the rule 7.5.1 [16], meaning they must be in the correct position in the moment of the serve. In order to identify the rotation, the position of the setter is considered the reference (i.e., when the setter is in zone 1, the team is in position 1 (P1)). All these factors make the structure of volleyball quite different amongst rotations, which turns them into a crucial variable when studying the game [18].

With all this in mind, some questions arise. Are the top male and female European volleyball teams making use of the space of play similarly? Can the distances or the trajectories of the setters determine the performance of their teams? How do variables such as the rotation or the attack lane affect the side-out success?

Therefore, the aims of this study were: (1) to compare the SARA between male and female high-level volleyball; and (2) to determine the relationship between variables such as distances, trajectories, the rotation of the team, and the attack lane and their influence in the success of the side-out in high-level male and female volleyball.

## 2. Methods

### 2.1. Sample

A total of 1302 side-outs from the 2019 European Volleyball Championships were registered (639 male, 663 female). The male European Championship took place in September (12th–29th) in France, Belgium, Slovenia, and the Netherlands, and the female European Championship took place from August 23rd to September 8th in Turkey, Poland, Hungary, and Slovakia. The first four classified teams of each championship were analyzed: Serbia, Slovenia, Poland, and France (male); Serbia, Turkey, Italy, and Poland (female). The matches corresponded to the last three games of each team in the championship (quarter-finals, semi-finals, and gold/bronze medal match). Data were only registered when the setter performed the setting action in the side-out phase; side-outs where another player set were not considered.

The study was conducted under approval of the Ethical Committee for Human Research of the University of Granada for the project with reference DEP2011-27503.

### 2.2. Variables

**Figure 1:**
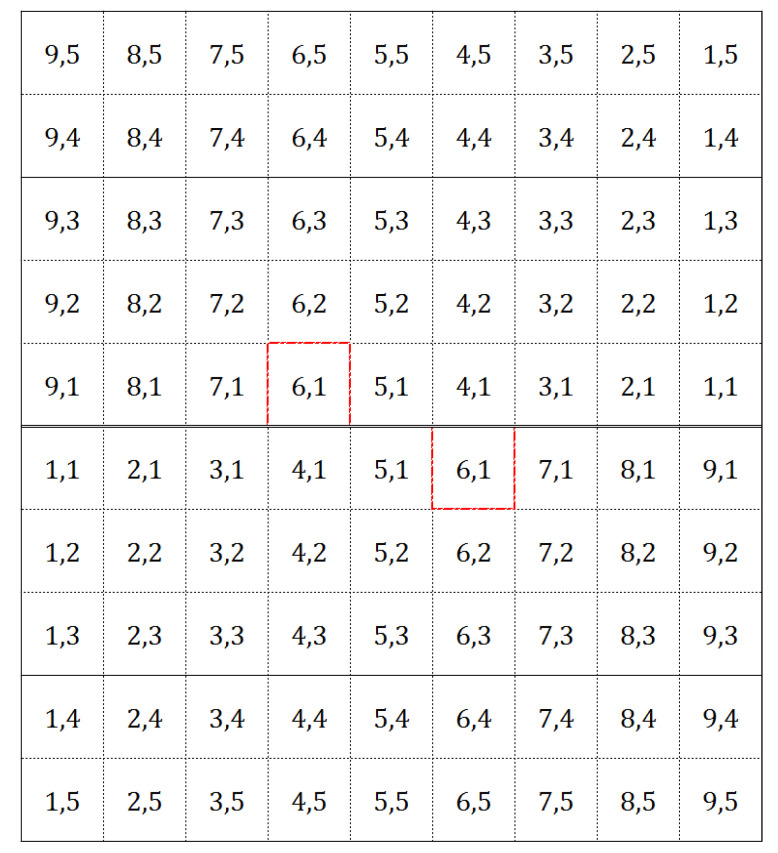
Latitude and depth (respectively) of the setter, and ideal setting zone (in red).

In order to determine the position of the setter, his or her latitude (from 1 to 9, comprising the nine meters in width of the volleyball court) and depth (from 1 to 5, comprising the five closest meters to the net) were registered. Each cell of the grid represents 1 × 1 m (one square meter; Figure 1). The position was registered both in the moment of the first contact (latitude of the setter in the moment of reception (*LR*) and depth of the setter in the moment of reception (*DR*)) and in the moment of the set (latitude of the setter in the moment of set (*LS*) and depth of the setter in the moment of set (*DS*)). The “moment of reception” was considered as the exact frame when the first contact was performed. The “moment of set” was considered as the exact frame when the setter contacted the ball (second contact). The following criteria were considered:

(a) If both feet were on the ground, the nearest to the ideal setting zone (latitude 6 and depth 1) was considered.
(b) If only one foot contacted the ground, that foot was the one to consider.
(c) If the foot to consider was between two cells, the nearest to the ideal setting zone was registered.
(d) If only one foot was visible, that foot was the one to consider; if no feet were visible, the cell corresponding to the projection of the body was registered.
(e) For the moment of set, if there was a jump set, the last contact with the ground before the jump was considered.

The average position of the setter in the moment of reception (APR) and the average position of the setter in the moment of set (APS) were calculated according to Formula (1), which represents the expression for the determination of the position (*c_x_*, *c_y_*), where *gd* is the maximum depth of the grid (5) and *gl* is the maximum latitude of the grid (9). Then *i*, *j* corresponds to each of the cells that compose the grid in depth (*i*) and latitude (*j*) and *f_i,j_* is the relative frequency associated with each grid position where the setter is in the total of the team matches. The APR and APS represent the mean latitude and depth of the setter (location on the court) in the moments of reception and set, as described above. The distance from the APS to the ideal setting zone (Distance 1) and from the APR to the APS (Distance 2) were calculated according to Formula (2), which represents the calculation of the Euclidean distance between two points, *c* and *p*, in the geometrical plane. The ideal setting zone was considered as the center of the most successful and frequent setting zone [7,19] (latitude 6 and depth 1; Figure 1).

(1) $$\left(c_{x},\;c_{y}\right)=\;\overset{gd}{\underset{i=1}{\sum }}\overset{gl}{\underset{j=1}{\sum }}\left(i\cdot f_{i,\;j},\;j\cdot f_{i,j}\right)$$

(2) $$\rho =D\left(c,p\right)=\sqrt{{\left|c_{x}-p_{x}\right|}^{2}+\;{\left|c_{y}-p_{y}\right|}^{2}}$$

**Figure 2:**
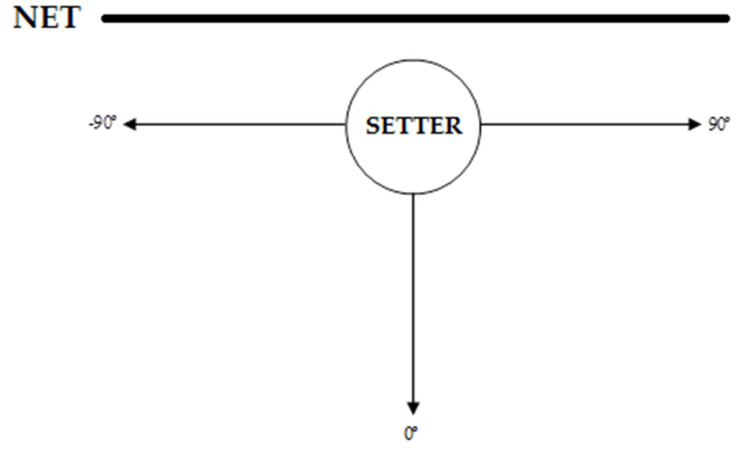
Calculation of the angle from the position in the moment of the reception to the position in the moment of the set.

The angle described in the trajectory between APR and APS (ARS) was calculated as Figure 2 explains; the “setter” represents the moment of reception, whereas the arrows represent the moment of set (i.e., a movement towards the right side of the court, completely parallel to the net, from the moment of reception to the moment of set, means an angle of 90°).

The attack lanes (AL) were described as right (attack taking place in the right two meters of the court), central (attack performed in the central five meters of the court), and left (attack carried out in the left two meters of the court), following the criteria by Sapena Peiro, Parra, Leon, Fradua, Benitez, and Urena [15]. The availability of first tempo was registered as a yes/no variable, considering whether the middle blocker presented the intention to attack both in timing and space [4]. Likewise, the result of the side-out (point or no point) and the rotation of the team in reception (P1, P2, P3, P4, P5, or P6) were registered. For the rotation, the position of the setter was considered, as the official regulations establish [16]. The reception efficacy was collected from the Confédération Européenne de Volleyball (CEV) website (https://www.cev.eu). It was calculated as the ratio between the number of excellent receptions and the total number of receptions.

### 2.3. Design, Instruments, and Reliability

The data were collected following the observational methodology. A nomothetic, punctual, and multidimensional observational design was used [20].

The matches were recorded with a camera located between 5 and 10 m from the back of the court. The videos were recorded with a resolution of 1280 × 720 pixels per inch and a frame rate of 30 frames per second. The position of the setter was registered utilizing the Kinovea software (version 0.8.24. www.kinovea.org; open source software, Boston, MA USA), specifically the adjustable grid, which was used as a reference [21]. The registration was conducted in a separate ad hoc Microsoft Excel spreadsheet.

Two different observers were trained in order to achieve consistency and reliability in the data gathering. For purposes of reliability of the observations, a 10% of the sample was randomly selected [22] and a Cohen’s Kappa [23] was carried out. The values of inter-observer and intra-observer reliability are shown in Table 1, which ranged between 0.914 and 1, thus being above the recommended 0.75 [24].

### 2.4. Data Analysis

A descriptive study of the data was carried out (frequencies and percentages). The Pearson correlation coefficient was used in order to determine the association between the ranking and Distance 1. Significance was set at *p* < 0.05. The library that implements the Recursive Partitioning for classification, regression and survival tree (RPART) model was used (https://cran.r-project.org/web/packages/rpart/index.html), with a training and test data set of 80% and 20% respectively. The RPART classification and regression trees (CART) implementation using the default values was applied to process the data and obtain the predictions. The accuracy was defined as the percentage of correctly classified instances (TP + TN)/(TP + TN + FP + FN), where TP, FN, FP, and TN represented the number of true positives, false negatives, false positives, and true negatives, respectively. All the implemented measures and metrics such as distances, average positions or angles have been calculated using the R software (http://www.r-project.org), in addition to the visualizations through our volleyball performance analysis (VPA) package (see link https://github.com/manuparra/volleyball-performance-analysis).

## 3. Results

The descriptive data (Table 2) showed that, in the male category, the largest Distance 1 corresponded to Serbia (2.033 m) and the smallest to France (1.288 m), whereas for Distance 2 the largest was of Slovenia (1.758 m) and the smallest of Poland (1.133). Serbia presented the lowest reception efficacy (0.19) and the rest of the teams’ efficacies were 0.33. In the female category, the largest Distance 1 and Distance 2 corresponded to Italy (1.494 m and 1.162 m, respectively), whereas the smallest Distance 1 was of Turkey (1.275 m) and the smallest Distance 2 was of Serbia (1.014). Turkey had the highest reception efficacy (0.43) and Poland the lowest (0.23).

**Figure 3:**
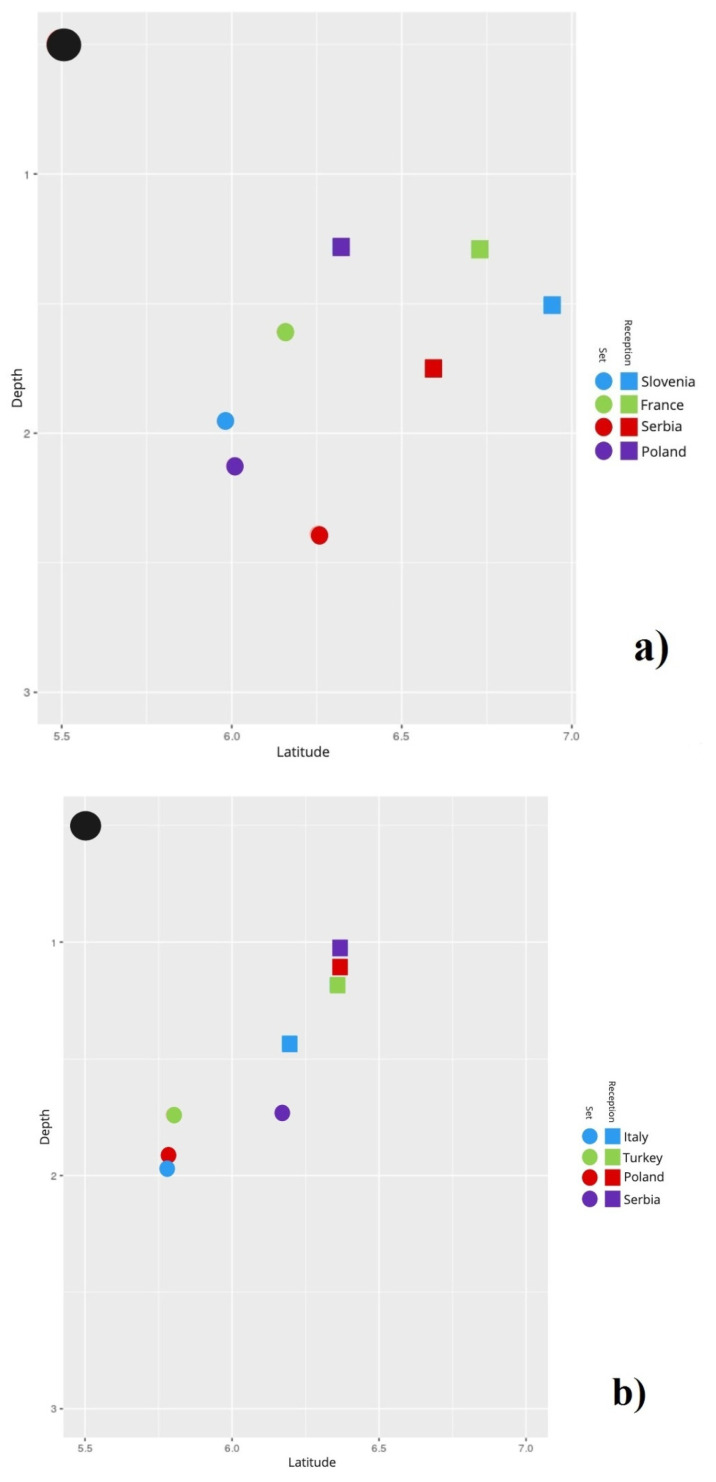
Average position of the setter in the moment of reception and average position of the setter in the moment of set of the male (**a**) and female (**b**) national teams.

The APS and APR of the male (a) and female (b) teams are presented in Figure 3. The Pearson correlation coefficient showed a non-significant value of *r* = 0.85 (*p* = 0.1492) between the variables Ranking and Distance 1.

The descriptive data concerning side-out and first tempo availability frequencies are presented in Table 3.

**Figure 4:**
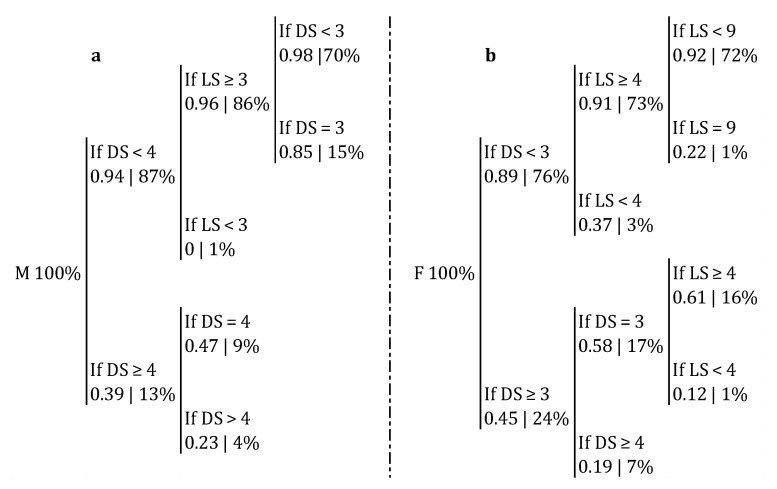
Probability of first tempo availability in male (**a**) and female (**b**) category depending on the LS and the DS. M: male category; F: female category; LS: latitude of the setter in the moment of set; DS: depth of the setter in the moment of set.

**Figure 5:**
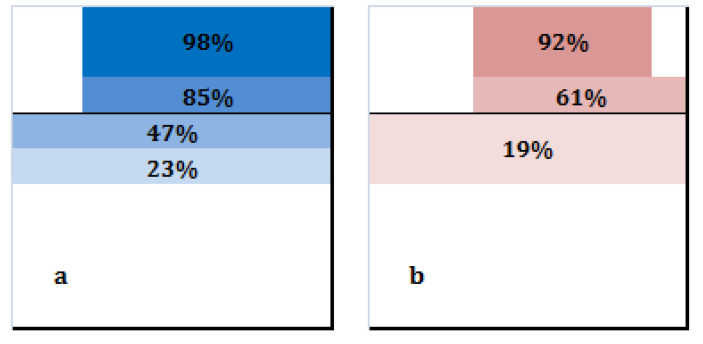
Probability of first tempo availability in male (**a**) and female (**b**) category according to the position of the setter in the moment of the set.

In order to determine differences between male and female teams in first tempo availability according to the position of the setter in the moment of the set, a classification tree for each category was built (Figure 4). In the male category, there was a probability of 0.98 of availability of first tempo in side-out when the setter performed in the two closest meters to the net and in a latitude between 3 and 9. If the setter performed in a depth of 3, the probability decreased to 0.85. If he moved further from the net, to the fourth and fifth meter and regardless of the latitude, this probability kept decreasing to 47% and 23%, respectively (Figure 5a). The classification accuracy was 0.899 (*p* < 0.001). In the female category, there was a probability of availability of first tempo of 0.92 if the setter performed in depth 2 or less and in latitude between 4 and 8. If the set took place in the third meter (depth 3) and the latitude was 5 or more, the probability decreased to 0.61; and if it happened in depth 4 or more, regardless of the latitude, it decreased to 0.19 (Figure 5b). The classification accuracy was 0.802 (*p* < 0.001).

**Figure 6:**
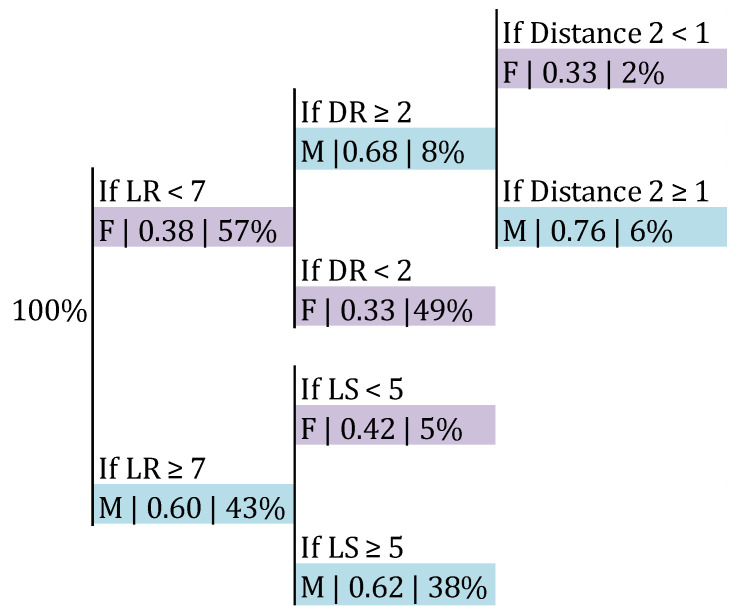
Probability of Category depending on the LR, DR, LS and DS and Distance 2. LR: latitude of the setter in the moment of reception; DR: depth of the setter in the moment of reception.

A classification tree was built in order to discriminate between male and female volleyball according to the position of the setter and Distance 2 (Figure 6). With a probability of 0.62, it was the male category if the LR was 7 or higher and the LS was 5 or higher. The classification accuracy was 0.716 (*p* > 0.05).

**Figure 7:**
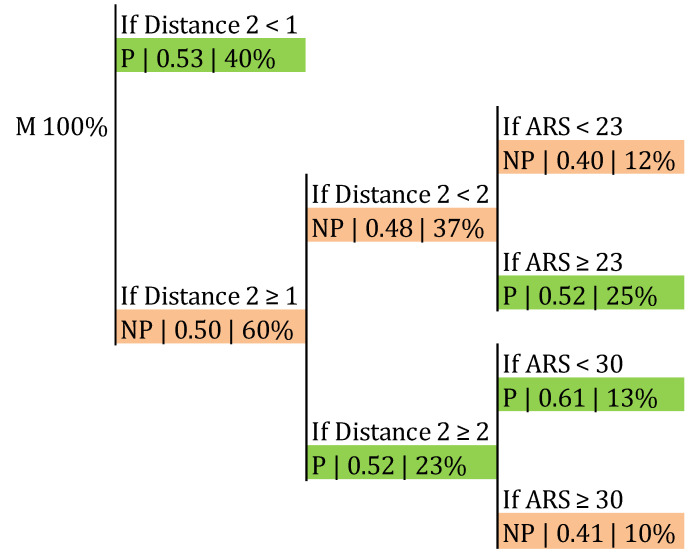
Probability of a point in male category in the side-out depending on Distance 2 and the ARS. ARS: angle described in the trajectory between APR and APS; P: point; NP: no point.

**Figure 8:**
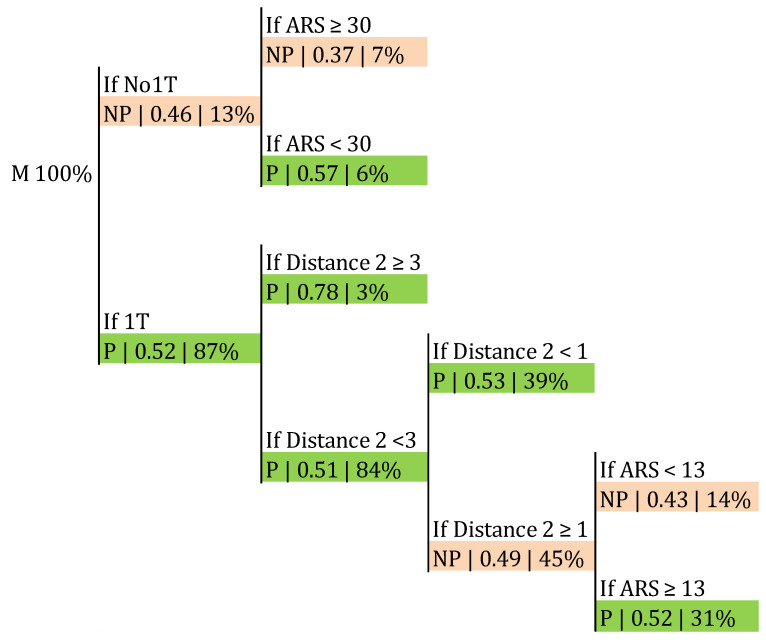
Probability of a point in the side-out depending on the availability of first tempo, Distance 2, and ARS.

Within the male sample, the frequency of success in the side-out was 50.57%, as aforementioned (Table 3). The classification trees presented in Figure 7 and Figure 8 allowed us to check if this frequency was affected by Distance 2, the ARS, and the first tempo availability. The probability of a point in the side-out increased to 0.61 when the ARS was lower than 30° and Distance 2 was 2 m or higher (Figure 7). The classification accuracy was 0.687 (*p* > 0.05). Furthermore, this probability grew to 0.78 if that distance increased to 3 or more meters, provided there was first tempo availability and regardless of the angle (Figure 8). The classification accuracy was 0.665 (*p* > 0.05).

**Figure 9:**
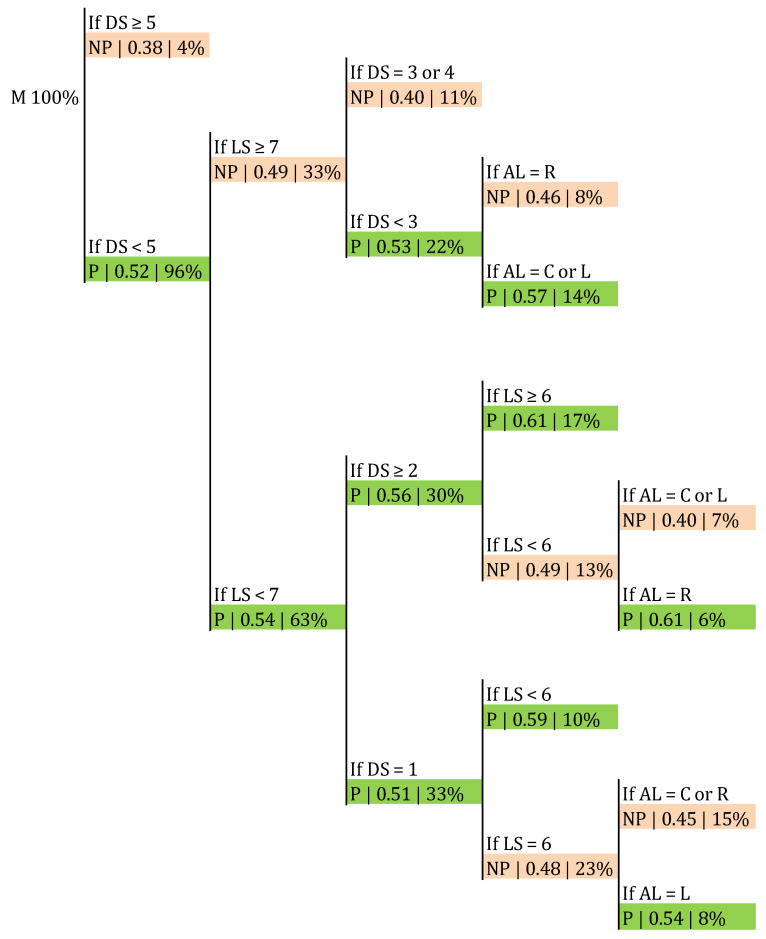
Probability of a point in the side-out depending on the attack lane, the LS, and DS; R: right attack lane; C: central attack lane; L: left attack lane.

The influence of the AL in the result of the side-out according to the LS and DS is shown in Figure 9. When the setter performed in a latitude of 5 or lower and in a depth of 2 or more, if the set went to the right lane there was a 0.61 probability of a point in side-out. The classification accuracy was 0.684 (*p* > 0.05).

**Figure 10:**
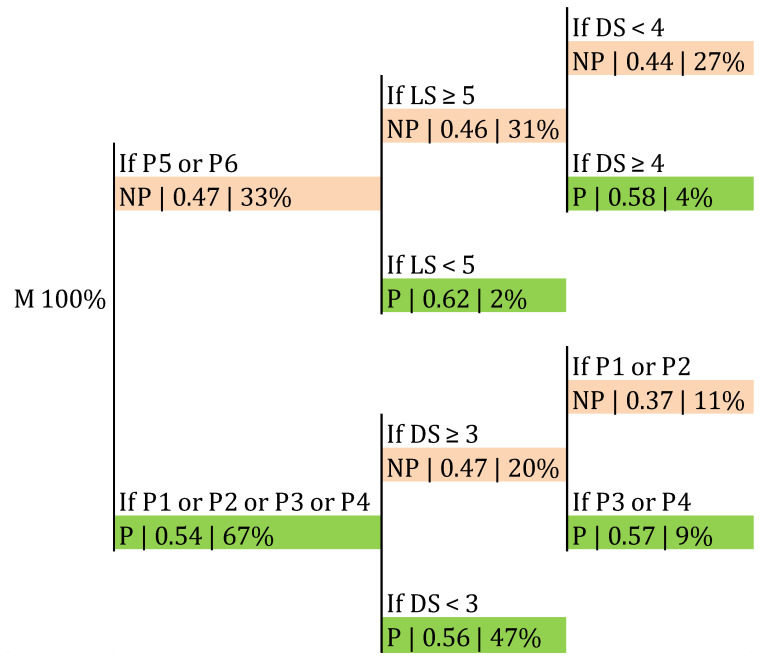
Probability of a point in the side-out depending on the Rotation, the LS and DS. P: rotation.

Concerning the effect of the rotation, the LS and the DS on the side-out success, results are shown in Figure 10. There was a probability of 0.56 of a point in the side-out in P1, P2, P3, and P4 when the DS was lower than 3. On the other hand, this probability was similar (0.57) when the DS was 3 or higher, but only in P3 and P4. For P5 and P6, if the LS was lower than 5, the probability of success in the side-out was 0.62 (Figure 10). The classification accuracy was 0.611 (*p* > 0.05).

## 4. Discussion

One of the aims of this study was to compare the SARA between male and female teams. Another objective was to determine, if any, the possible associations between the rotation of the team, the different distances covered by the setter, the angle described in that trajectory and the attack lane, and their influence in the result of the side-out in male and female teams.

Concerning reception efficacy, Lima, Palao, Moreira, and Clemente [14] concluded that the different height of the net in the female competition could explain their lower reception efficacy. However, both our spatial results and the statistics collected from the CEV tell us otherwise. In this sample, women presented a higher reception efficacy and smaller Distances 1 and 2 (Table 2), which agrees with the fact that male setters tended to perform in larger areas than female setters (Figure 3). Because of the lower quality of their receptions, male teams might be evolving their game through expanding the area in which the middle blocker is available for first tempo attacks [7] (Figure 4). A possible explanation of this lower reception efficacy and efficiency in male teams could be the evolution of the jump serve, which is the fastest, riskiest, most effective, and most common type of serve, whereas in women is not as frequent and powerful [25,26]. According to Lima, Palao, Moreira, and Clemente [14], male teams might be trying to decrease the side-out chances of the opponent by taking more risks (i.e., more errors but also more points). As a consequence, the break points are acquiring great relevance [13]. Nevertheless, it seems the jump serve only affects the reception, given that the setting efficacy is not altered by any serve-related variable [10]. Therefore, it seems that male teams are managing to adapt their reception in a spatial fashion rather than through the increase of the traditional reception performance, without impairing their setting results.

Another interesting issue concerns the relationship between side-out result and availability of first tempo. Male teams presented a side-out success of 52.08% and 41.57% with and without first tempo availability, respectively, which means a variation of 10.51%. On the other hand, female teams presented a 49.44% of success with middle blocker availability and 31.79% without it, which represents a difference of 17.65% (Table 3). These results confirm the relevance of being able to play with the middle blocker in the side-out [27]. Furthermore, female teams decreased their first tempo availability more as their setters performed further from the net than male teams (Figure 5), meaning that they were able to play first tempo attacks with a lower probability in certain areas of intervention of their setters. When male setters performed 3 m away from the net, their teams presented a probability of first tempo availability of 85%, whereas for female teams this probability was 61%. In DS 4 and 5, male teams had a 47% and 23% probability of first tempo, respectively, whereas female teams presented a probability of 19%. As aforementioned, females’ Distances 1 were smaller and reception efficacies were higher than males’. This fact could be making it unnecessary for them to enlarge the space where they are able to play first tempo attacks. Along the same lines, our results have shown smaller Distances 2 for women than for men. This means that women covered, on average, less distance from the moment of the reception until the moment of the set. For example, as Figure 6 explains, when Distance 2 was 1 m or bigger, there was a probability of 0.76 of dealing with the volleyball in males. A possible explanation for these differences in the usage of the space might have to do with the dissimilar physical abilities between men and women [28,29], which could be preventing female players to make the most of the space of play. On a similar note, younger players are usually assigned specific playing positions that require them to perform actions which might be exceeding their physical abilities (i.e., a U-14 setter who is required to set the ball far from the net instead of allowing a teammate who is not responsible for that action to do it).

Concerning Distance 1 of the male teams, for Mercado-Palomino, Millán-Sánchez, Parra-Royón, Benítez, and Ureña Espa [7], Distance 1 correlated positively with the ranking in their study with a World Championship sample from 2010. Our results show no statistical association in this regard. Thus, with this sample it cannot be confirmed that Distance 1 is associated to the ranking, and therefore we cannot state that there are significant differences between the teams. A possible explanation could be that what was a performance indicator in 2010 has now become a general trend that is not different between teams. Notwithstanding, the small sample size of our study may decrease the statistical power, and as a consequence, this interpretation should be considered with caution. Nevertheless, addressing exclusively the descriptive data, Mercado-Palomino, Millán-Sánchez, Parra-Royón, Benítez, and Ureña Espa [7] obtained values between 1.1687 m and 1.4675 m in the top four classified teams. Our results regarding the top four ranked teams in the European Championship of 2019 presented Distances 1 between 1.288 m and 2.033 m. These results might be showing that male teams are able to play first tempo attacks from areas further from the ideal setting zone, which represents an evolution throughout the last decade. Furthermore, the national teams of Serbia and France participated in both championships; Serbia evolved from a Distance 1 of 1.3577 m in 2010 to 2.033 m in 2019, whereas France increased its Distance 1 from 1.0491 m to 1.288 m, confirming this trend not only in the average of the teams, but also in particular cases.

Concerning the angle, according to our results (Figure 7) male teams increased their side-out success to 0.61 when the ARS was lower than 30°, provided that Distance 2 was 2 m or more. This means that there was a higher probability of a point in the side-out when male setters moved 2 m or more and frontwards or slightly backwards (ARS < 30°; Figure 2). We have not found previous research measuring the distance or trajectory covered by the setter before setting; nevertheless, Silva, et al. [30] found that in P4 and P5 the game was slower and more predictable, being a possible explanation that in these rotations the distances for the setter in the side-out were longer. According to our findings, in general, higher distances meant better results (Figure 8). According to this result, the training of the reception could be focused on encouraging the players responsible for that action to send the pass in front of the setter, avoiding his or her movement backwards.

Previous studies have tried to predict the attack zone. In a U-16 Spanish male sample, the reception zone (*p* = 0.007) and the reception efficacy (*p* < 0.001) predicted what the attack zone was going to be [31]; when receiving in zone 1, the set tended to go to zone 2, and when receiving in zone 6, the set tended to go to zone 4. Furthermore, when the reception efficacy was not good, the set went to the back row, when it was good, the set went to 2 and 4, and when it was excellent, the set went to 3. Thus, it seems that some quality in the first contact is required in order to play with the middle blockers in U-16 male players. Our results did not consider the relationship between reception efficacy and attack zone, but the association between the position of the setter and the AL, all related to the probability of success in high-level volleyball for males. It seemed that when the setter performed in the left half of the court (latitude 5 or less) and not in the first meter from the net (depth 2 or more), if he set the ball to the right lane (zones 1 or 2), the probability of a point increased from the overall average of 50.57% to a 61%, meaning if he set to his back when he was moving frontwards and away from the net (Figure 9). This finding could encourage coaches to generate situations in their training processes where the players responsible for the first contact avoid passing the ball exactly where the setter is located, but making him or her move a little instead, in order to create some sort of advantage.

With regard to the rotations, previous studies have found that P1, P6, and P4 were the ones which contributed more to winning matches overall [30,32]. In our study, the result of the match was not considered; however, we found that, depending on the position of the setter in the moment of the set, some rotations might be more likely to be successful in the side-out. The probability of a point in the side-out increased with respect to the average in P1, P2, P3, and P4 if the DS was lower than 3. Furthermore, for P5 and P6, the probability of success was 0.62 if the LS was lower than 5 (Figure 10). These results concerning the rotations might be affected by other factors not included in our analyses, such as the number of front attackers or the attacking variability in female teams (i.e., slide attack) [33], which represents a limitation of this study.

These results provide an important insight on the availability of first tempo in high-level teams. According to our findings, elite teams seek to develop their game by maintaining the possibility to play quick attacks despite the position of the setter far from the traditionally optimal. When assessing their performance, the teams should consider the ability to play quick attacks when their reception is not as precise as they would expect. Given the importance of this variable, coaches should consider working towards an increase of the SARA in their teams, and by doing so improving their performance in the side-out. Besides, these findings concerning the trends of the elite level might encourage coaches of younger teams to compare themselves and, by doing so, to guide their training processes towards such trends.

This work presents some limitations. Only three matches of each team were analyzed. Therefore, the average position of the setter is calculated considering only those three matches. Nevertheless, the reception efficacy, as extracted from the official statistics of the European Championship, comprises data from all nine matches played by the teams throughout the whole tournament, which might represent interference. Additionally, the small sample size might be reducing the statistical power. All these factors suggest a cautious interpretation of the results, meaning it might not be fully appropriate to generalize. Moreover, in order to determine the full influence that specific aspects (i.e., the setter’s action range with first tempo availability) belonging to a whole (side-out) might have, it is necessary to study it as a whole. This study has not considered aspects such as the opponent serve, the number of available attackers, or the receiver player.

## 5. Conclusions

Our results have shown that female teams seem to present more reduced SARAs than male teams, according to their higher reception efficacies and smaller Distance 1 and Distance 2, meaning female setters tend to play closer to the net than male setters. The correlation between the ranking and Distance 1 in high-level male teams was not statistically significant, which leads us to interpret that the ranking does not depend on Distance 1 and the teams in this sample do not differ extraordinarily amongst them. Lastly, it seemed that a movement of at least one meter (or more depending on the circumstances) frontwards or slightly backwards (less than 30°) might improve the performance of the side-out.

This work provides information on a quite recent measurement that assesses an aspect that is happening in the game and that might affect the performance. This measurement complements the information provided by the usual performance indicators.

Future studies should include the analysis of other factors influencing the side-out phase, in order to contextualize the offensive actions of the teams properly. Likewise, the analysis of the SARA in younger categories, as well as non-professional players, might be an interesting avenue of research, in order to compare the results to those of the elite-level players.

## Figures and Tables

**Table 1:** Inter-observer and intra-observer reliability.

Cohen’s Kappa	Inter-Observer	Intra-Observer
Team	1	1
Setter’s position	1	0.914
Availability of first tempo	1	0.99
Side-out result	1	1
Attack lane	0.988	0.988
Team rotation	0.981	1

**Table 2:** Reception efficacy, APS and APR coordinates (latitude and depth; mean ± standard deviation), Distance 1, Distance 2, and ARS of the top four classified teams in each category.

TEAM		Reception Efficacy	APS Coordinates (Lat, Dep)	Distance 1 (m)	APR Coordinates (Lat, Dep)	Distance 2 (m)	ARS (°)
M							
	Serbia	0.19	6.253 ± 0.695, 2.388 ± 0.169	2.033	6.593 ± 0.972, 1.753 ± 0.347	1.663	−34.25
	Slovenia	0.33	5.980 ± 0.953, 1.953 ± 0.121	1.531	6.941 ± 1.180, 1.507 ± 0.246	1.758	−71.53
	Poland	0.33	6.007 ± 1.001, 2.125 ± 0.190	1.702	6.320 ± 1.179, 1.261 ± 0.321	1.133	−26.76
	France	0.33	6.156 ± 0.762, 1.608 ± 0.217	1.288	6.729 ± 1.309, 1.289 ± 0.321	1.460	−67.33
F							
	Serbia	0.37	6.169 ± 0.929, 1.730 ± 0.199	1.401	6.368 ± 1.120, 1.023 ± 0.432	1.014	−19.44
	Turkey	0.43	5.800 ± 0.824, 1.739 ± 0.193	1.275	6.358 ± 1.163, 1.182 ± 0.374	1.096	−51.35
	Italy	0.30	5.778 ± 0.789, 1.968 ± 0.199	1.494	6.195 ± 0.893, 1.432 ± 0.333	1.162	−45.26
	Poland	0.23	5.782 ± 0.762, 1.914 ± 0.198	1.442	6.368 ± 1.079, 1.105 ± 0.394	1.059	−42.56

M: male category; F: female category; APS: average position of the setter in the moment of the set; APR: average position of the setter in the moment of the reception; Lat: latitude; Dep: depth; ARS: angle of the trajectory of the setter from the moment of the reception to the moment of the set.

**Table 3:** Frequencies of the side-out and first tempo availability.

	Total Side-Outs (Side-Out Points; % Of The Total Side-Outs)	Side-Outs with 1T Availability (% of the Total Side-Outs)	Side-Outs without 1T Availability (% of the Total Side-Outs)	Side-Out Points with 1T Availability (% of the Side-Outs with 1T Availability)	Side-Out Points without 1T Availability (% of the Side-Outs without 1T Availability)
MALE	619 (313; 50.57)	530 (85, 62)	89 (14, 38)	276 (52, 08)	37 (41, 57)
FEMALE	683 (311; 45.53)	532 (77, 89)	151 (22, 11)	263 (49, 44)	48 (31, 79)
TOTAL	1302 (624; 47.93)	1062 (81, 57)	240 (18, 43)	539 (50, 75)	85 (35, 42)

1T: first tempo.
